# Autoantibody Positivity in Two Bahraini Siblings With a Novel Alpha-Methylacyl-CoA Racemase Mutation

**DOI:** 10.7759/cureus.41720

**Published:** 2023-07-11

**Authors:** Hasan M Isa, Ahmed D Khudair, Rachel A Marshall, Aiman D Khudair, Thuraiya H Al-Rawahia, Maryam Y Busehail

**Affiliations:** 1 Department of Pediatrics, Arabian Gulf University, Manama, BHR; 2 Department of Pediatrics, Salmaniya Medical Complex, Manama, BHR; 3 Department of Pediatrics, Royal College of Surgeons in Ireland - Bahrain, Muharraq, BHR

**Keywords:** bile acid synthesis disorders, auto antibodies, child, alpha-methylacyl-coa racemase, whole exome sequencing, bile acid synthesis defect, bahrain

## Abstract

Bile acid synthesis disorders (BASD) are a group of rare autosomal recessive disorders. Of the nine different versions, BASD type 4 is characterized by a gene mutation in alpha-methylacyl-CoA racemase (AMACR), which is located on chromosome 5p13. These disorders generally present with a normal gamma-glutamyl transferase with cholestasis, absence of pruritis, and malabsorption of fat, which can lead to fat-soluble vitamin deficiencies. In adulthood, patients usually develop neurological sequelae. Initial testing can be done through the measurement of urine metabolites; however, confirmation of the diagnosis is achieved through whole exome sequencing. Treatment involves supplementation of oral cholic acid and modification of diet. Only 23 patients with this disease have been described. Here, we report two cases of siblings from a family in Bahrain with a novel AMACR mutation and a unique association with autoimmune antibodies alongside a literature review.

## Introduction

Bile acid synthesis disorders (BASD) are a group of rare autosomal recessive disorders that can be classified into nine different variations based on the gene mutated [1]. These patients suffer from symptoms related to bile acid insufficiency and the consequences of its toxic intermediary backup in the body [1]. Clinical findings include normal gamma-glutamyl transferase (GGT) with cholestasis, absence of pruritis, as well as lipid and fat-soluble vitamin malabsorption [1]. Hepatic failure will ensue if these disorders are not detected early [2].

BASD type 4 is characterized by an alpha-methylacyl-CoA racemase (AMACR) gene mutation on chromosome 5p13 [3]. Normally, the AMACR enzyme partakes in the racemization of trihydroxycholestanoic acid (THCA) and pristanic acid stereoisomers. This conversion is required in the process of peroxisomal beta-oxidation of branched-chain fatty acids and their intermediates. Deficiency of this enzyme leads to the buildup of dihydroxycholestanoic acid, THCA, and pristanic acid in the plasma [1,4,5].

Clinically, patients with AMACR deficiency usually present with neonatal cholestasis and fat-soluble vitamin deficiencies. Neurological sequelae, such as seizures and peripheral neuropathy, can also emerge as manifestations of this condition in adulthood [1,3]. Laboratory investigations typically reveal an elevation in serum pristanic acid and THCA levels, along with normal levels of long-chain fatty acids and phytanic acids, as well as a decrease in cholic acid and chenodeoxycholic acid, which are the primary bile acids found in the body [1,6].

This report presents two Bahraini siblings, a five-year-old girl and an 11-year-old boy, with AMACR deficiency and autoimmune antibody positivity. These are the first two cases of AMACR deficiency to be reported in Bahrain.

## Case presentation

Case 1

**Figure 1:**
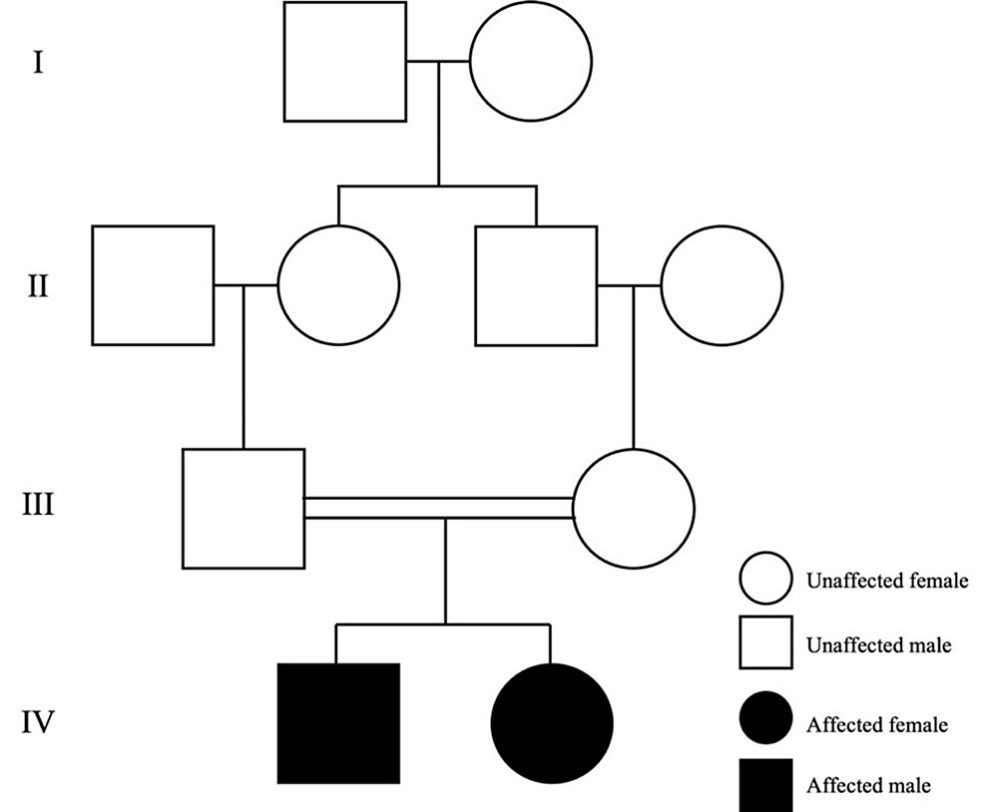
Family pedigree of two siblings with a novel alpha-methylacyl-CoA racemase mutation with consanguineous parents.

The first patient was a five-year-old Bahraini girl. She was born via cesarean section at term to consanguineous parents who are first cousins (Figure 1). Her appearance, pulse, grimace, activity, and respiratory scores were 9 and 10 at one and five minutes, respectively, with a birth weight of 2.9 kilograms (kg).

She was born with a down-slanted palpebral fissure and a cleft palate that was corrected with surgery. Other details regarding her birth were unremarkable. The patient was up to date with all her vaccinations. There was no relevant family history related to liver or metabolic disorders.

The patient first presented in Iran at six months of age after concerns from her parents that she was lethargic compared to other children and had a loss of appetite. She was subsequently lost to follow-up; however, she later presented again in Iran for the same symptoms at the age of five years. Liver function tests (LFTs) indicated elevated levels of aspartate aminotransferase (AST) of 86 U/L (normal range: <47) and alanine aminotransferase (ALT) of 119 U/L (normal range: <40). Biochemical testing showed high antinuclear antibodies (ANA) of 1.83 (index; negative < 0.8, equivocal = 0.8-1.2, positive > 1.2). Abdominal ultrasound showed a fatty liver of a normal size. Following these results, a liver biopsy was requested to rule out autoimmune hepatitis (AIH), and she was referred to Bahrain to conduct further testing. No other interventions were implemented at this time.

Within the same year, she presented to the pediatric gastroenterologist in Bahrain as she was still experiencing lethargy and poor appetite. Additionally, she had a protruded abdomen and right upper quadrant (RUQ) pain. She weighed 17.1 kg and was 103 centimeters (cm) tall with a body mass index (BMI) of 16.1 kg/m^2^. The patient was in the 33^rd ^percentile for weight and 9^th^ percentile for height according to the World Health Organization growth chart reference. On physical examination, she was pink, active, and anicteric. Her abdomen was slightly distended with gases, but there was no hepatosplenomegaly or ascites. Eye examination was unremarkable, with no Kayser-Fleischer rings. She underwent investigations to rule out viral hepatitis and other liver disorders such as Wilson's disease, AIH, and metabolic disorders. LFTs revealed a high ALT of 110 U/L (Table 1).

**Table 1:** Initial laboratory investigations of two pediatric patients with alpha-methylacyl-CoA racemase deficiency in Bahrain.

Laboratory investigations	Reference ranges	Patient 1	Patient 2
White blood cells count (x10^9^/L)	3.6-9.6	11.41	6.1
Hemoglobin (g/dL)	12.0-14.5	12.29	13.07
Platelets (x10^9^/L)	150-400	338.2	298.7
Total protein (g/L)	64-82	70	72
Albumin (g/L)	38-54	45	47
Globulin (g/L)	15-30	25	25
Total bilirubin (umol/L)	5-21	6	6
Alkaline phosphatase (U/L)	150-480	132	191
Alanine aminotransferase (U/L)	<41	110	90
Gamma-glutamyl transferase (U/L)	<35	13	17
Prothrombin time (s)	10.7-13.9	13.3	12.2
Activated partial thromboplastin time (s)	25-43	22.2	22.8
International normalized ratio	0.61-1.17	1.16	1.06

Biochemical investigations showed positivity for lipemia, and blood tests reported a normal vitamin D level of 53 nmol/L (normal range: >50). Laboratory testing for hepatitis A, B, and C was negative. Serology tests reported positive Epstein-Barr virus (EBV) immunoglobulin G (IgG) antibodies and high anti-cytomegalovirus (CMV) IgG. Immunoglobulin M (IgM) was negative for both of these viruses, indicating past infections. Total immunoglobulin levels showed normal IgG of 9.19 g/L (normal range: 4.9-16.1), IgM of 0.816 g/L (normal range: 0.5-2), and immunoglobulin A of 1.18 g/L (normal range: 0.4-2). Anti-neutrophil cytoplasmic antibodies, celiac screen, and auto-liver serologies, including smooth muscle antibodies (SMA), liver/kidney microsomal-1 (LKM-1), and anti-mitochondrial antibodies (AMA), were all negative. Serum copper, urinary copper, and ceruloplasmin levels were within normal ranges. Repeat upper abdominal ultrasound results were unremarkable. The patient was admitted to the hospital and underwent a liver biopsy.

**Figure 2:**
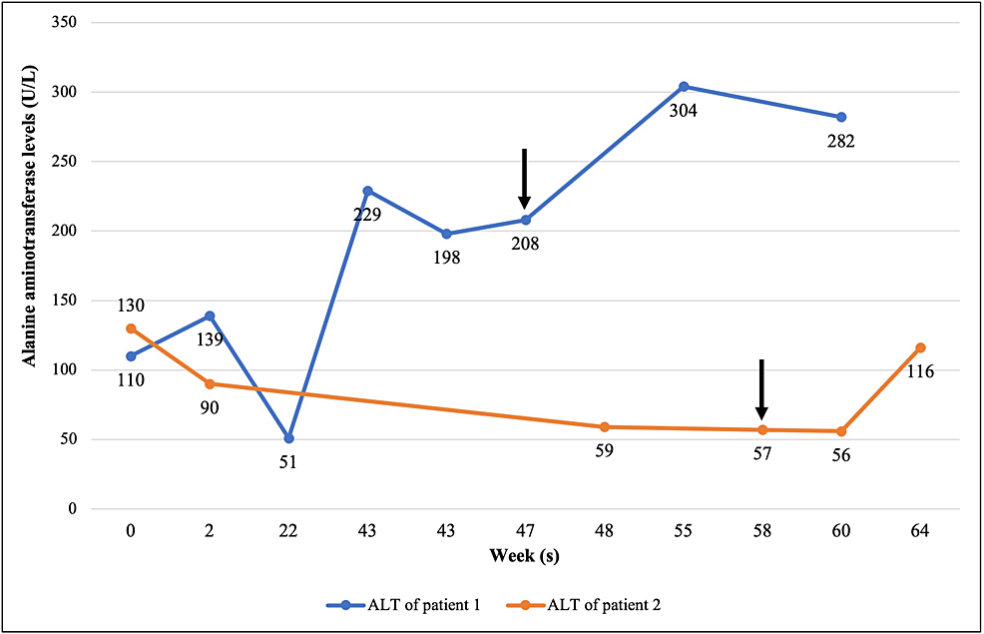
Alanine aminotransferase (ALT) levels of two patients with alpha-methylacyl-CoA racemase deficiency before and after administration of prednisolone (arrows). Normal alanine aminotransferase level is <40 U/L.

In view of positive ANA, the patient was started on intravenous methylprednisolone due to suspicion of the AIH. She was later discharged on oral prednisolone and asked to follow up. After three weeks, the patient returned to our clinic with no improvement in her LFTs and became irritable with a cushingoid appearance (Figure 2).

A liver biopsy with reticulin and Masson trichrome stains showed dense fibrosis of the portal triads without any significant inflammatory infiltrates. No giant cell formation was noted in the hepatocytes. The patient underwent repeat ANA testing, which was negative. Genetic testing was considered, and prednisolone was subsequently halted. Whole exome sequencing (WES) was performed and revealed a homozygous variant of uncertain significance mutation in the AMACR gene (c.553-3A>G). This confirmed a final diagnosis of BASD type 4. A frozen urine sample was submitted for special urine analysis and revealed normal concentrations of bile acid conjugates (glycine, taurine, sulfate, and glucuronide conjugates). She was advised to start treatment with cholic acid and was referred to a dietician.

Case 2

The second patient, an 11-year-old Bahraini boy, is the older brother of our first patient (Figure 1). He had an uneventful neonatal period and was up to date with all vaccinations. At 10 years of age, the patient was initially worked up due to obesity. He weighed 58 kg (>99^th^ percentile) and was 147.5 cm tall (94^th^ percentile) with a BMI of 26.7 kg/m^2^. Laboratory tests showed an elevated ALT level of 90 U/L and lipemia (Table 1). This elevation in LFTs was suspected to be due to nonalcoholic steatohepatitis (NASH). He was advised to lose weight through dieting and exercise. Later, the patient presented with his sister at her follow-up visit and underwent additional investigations as per request from the parents, despite him being asymptomatic. This was due to concern of a genetic disease following his sisters' failure to respond to steroids. He was pink and anicteric, and the systemic examination was unremarkable. Further laboratory investigations revealed a high ALT of 59 U/L. Serology tests were positive for EBV and CMV IgG but negative for IgM. Anti-SMA was positive. Hepatitis A, B, and C profile, ANA, anti-gastric parietal cell, anti-LKM-1 antibody, and AMA were all negative. Additionally, Wilson's disease workup was also negative.

An upper abdomen ultrasound was performed due to obesity with suspected fatty liver and NASH, deranged LFTs, and a family history of a sister with AIH. Liver ultrasound showed a mild diffuse increase in parenchymal echotexture with a normal size of 15 cm. The final impression was a normal-sized fatty liver. In view of the previous findings, the patient underwent a liver biopsy revealing mild inflammation of the portal tracts comprising mainly of lymphocytes, and macrovesicular steatosis of the centrivenular hepatocytes. The working diagnosis was AIH secondary to SMA positivity. Therefore, the patient was treated with oral prednisolone. However, a year later, after the patient suffered a bout of epistaxis, a liver panel was requested. His results showed a further elevation of ALT level of 116 U/L and positivity for lipemia and icterus (Figure 2). These clinical findings, along with the lack of response to steroid therapy, raised suspicion of genetic liver or platelet disorders.

The aforementioned events occurred two months following the diagnosis of BASD type 4 in his sister. He subsequently underwent carrier testing for the familial mutation that also revealed a homozygous variant of uncertain significance mutation in the AMACR gene (c.553-3A>G). This was the same variant as his sister, confirming a concordant diagnosis of BASD type 4. However, subsequent special urine analysis revealed normal concentrations of bile acid conjugates. Similar to his sister, the patient was referred to a dietician and advised to start treatment with cholic acid.

Both parents received genetic counseling, and carrier testing confirmed they were heterozygous for the same mutation. As of now, both patients have been developing normally with no neurological sequelae.

## Discussion

To date, there are only 23 previously documented cases of AMACR deficiency in the literature. In this report, we added two new cases. These are the first cases to be reported in Bahrain and the second set of cases within the Gulf Cooperation Council [7].

Patient sex is not an important factor in autosomal recessive diseases such as AMACR deficiency as it affects both genders equally. However, males were predominantly reported with this condition, comprising approximately 14 (61%) cases compared to females who only made up nine (39%). Patients who presented before the age of 18 accounted for nine (39%) of the cases, while those presenting during adulthood included 14 (61%) of the total cases. Our report presents an uncommon association of a female patient under the age of 18 with AMACR deficiency (patient 1).

Bahrain is a small country with a high rate of consanguineous marriage accounting for 11.4% in 2009 [8]. Our patients are siblings with identical AMACR mutations born to parents who are first cousins (Figure 1). Similarly, Alsalamah et al. and Haugarvoll et al. reported two families consisting of three and two siblings who shared the same AMACR mutation, respectively [7,9]. Being an autosomal recessive disease, consanguinity is expected to be a common pattern among parents of previously reported cases (n = 8, 35%).

**Figure 3:**
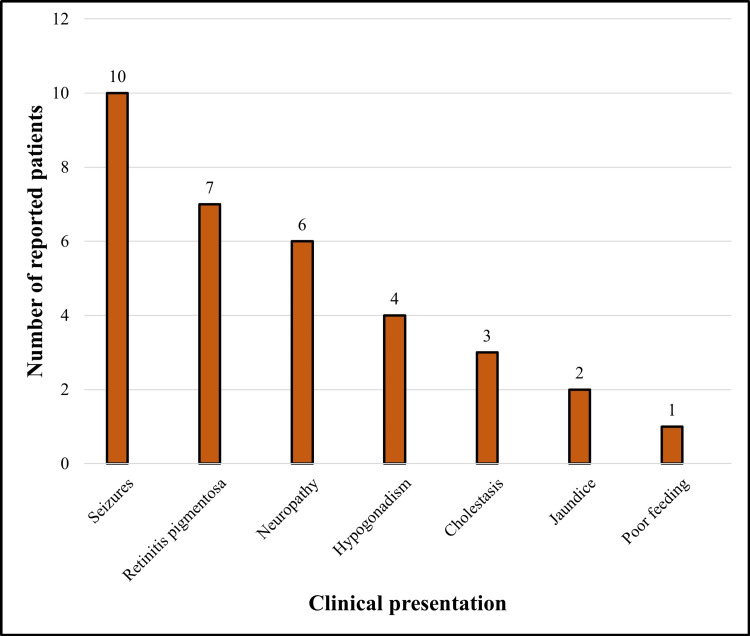
The prevalence of symptoms in previously reported alpha-methylacyl-CoA racemase deficiency cases.

The clinical presentation of this disease varies based on the patient’s age. In addition to lethargy, poor feeding was another presenting complaint in our female patient, which was only reported once by Saxena in a four-month-old male [10]. Other reported findings in pediatric patients that were not present in our case were neonatal cholestasis and jaundice [10,11]. Additionally, our female patient presented with previously unreported symptoms, such as protruded abdomen and RUQ pain. Our male patient was asymptomatic but was found to have an isolated elevation in liver enzymes via screening. This combination of findings is atypical in presentations of AMACR deficiency and has been reported once by Gündüz et al. in a 10-month-old boy [12]. Their patient was asymptomatic as well, except for serially elevated LFTs in infancy. However, contrary to our case, their patient presented with mild hepatosplenomegaly. Eventually, he was diagnosed with AMACR deficiency at the age of two years [12]. AMACR deficiency, if diagnosed late, usually presents with neurological and ophthalmological symptoms, most notably, seizures, neuropathies, and retinitis pigmentosa (Figure 3) [7].

Interestingly, Wang et al. reported a 71-year-old male with jaundice and the absence of seizures, which contradicts the general ideology that neurological symptoms are exclusive to adult patients with AMACR deficiency [2].

A diagnosis of BASD type 4 can be supported by a variety of diagnostic tools such as LFTs, pristanic and phytanic acid levels, and urine mass spectrometry [2,9,13]. The most prevalent findings in pediatric patients with AMACR deficiency are elevated pristanic acid levels, which were reported in 15 (67%) of the cases, as well as elevated LFTs in 10 (44%) of the cases [11,14-16]. Although we did not measure pristanic acid levels, LFTs were elevated in both of our patients. Other common findings in the pediatric population include fat-soluble vitamin deficiencies, which were previously reported by Alsalamah et al. and Setchell et al. [7,11]. In our case, patient 1 was investigated for vitamin D deficiency, but her level was normal.

In our report, urine metabolite testing showed normal levels of bile acid conjugates in both of our patients, which is inconclusive in diagnosing BASD type 4. Interestingly, we have not seen this association in previous literature, as those that used urine analysis all reported elevation in bile acid metabolites [11,14-16]. This presents a gap in the current understanding of AMACR deficiency diagnosis, as perhaps some mutations can present with metabolites in the urine, while some potentially do not [7,9,10].

A potentially underutilized method of objective measurement is retinal multimodal imaging and electrophysiology. Even in the absence of symptomatic retinal dysfunction, patients with AMACR mutations have an aberration in retinal function. This is a potential early marker of disease that can serve as a diagnostic clue pointing toward possible AMACR deficiency [7].

In both of our patients, the initial suspicion was AIH due to positive ANA in the girl and positive SMA in the boy. These markers are classically found in type 1 AIH [17]. As a result, prednisolone was administered after undergoing a liver biopsy, but both patients were unresponsive to the treatment (Figure 2). Moreover, on follow-up, repeated ANA testing in the girl revealed a negative result. This prompted a reevaluation of the diagnosis with a suspicion of an underlying genetic condition, which was confirmed to be BASD type 4. Upon review of the literature, this potential association between positive autoimmune antibodies and AMACR deficiency has not been seen before. This emphasizes the importance of considering the possibility of AMACR deficiency when reevaluating the diagnosis in a patient with AIH failing to respond to treatment.

On liver biopsy, the appearance of necrotic hepatocytes, giant cells, and intralobular cholestasis is potentially consistent with the diagnosis of BASD [18]. According to our knowledge, there have only been two liver biopsies done in patients with AMACR deficiency [11,14]. A biopsy reported by Setchell et al. found portal zone inflammation with hemosiderin-laden macrophages as well as multinucleated giant cells [11]. Another biopsy by Veldhoven et al. also found numerous giant cells and lymphocytic portal infiltrates; additionally, they noted that some portal tracts had no recognizable bile ducts [14]. In our female patient, a biopsy revealed dense fibrosis of portal triads. While our male patient’s biopsy showed mild portal tract inflammation, consisting mostly of lymphocytes, and macrovesicular steatosis around the centrivenular hepatocytes. Both biopsy results were not consistent with the general findings in BASD and other AMACR deficiency cases. This also shows a high rate of variability in biopsy findings, even within the same family members sharing the same mutation.

In both of our patients, genetic testing revealed a novel homozygous mutation in the AMACR gene, c.553-3A>G. This variant is predicted to disrupt a highly conserved acceptor splice site. It has been classified as a variant of uncertain significance (class 3), according to the recommendations of CENTOGENE and American College of Medical Genetics guidelines. To the best of our knowledge, the most common mutations formerly seen in patients with AMACR deficiency are c.154T>C in 10 cases (43%), c.877T>C in three cases (13%), and c.367G>A in two cases (9%) [7,11,16,19-25]. While the gene variants c.710A>G (4%), c.596G>A (4%), c.559G>A (4%), and c.320T>C (4%), were all reported once each [2,12,20,26]. There were four (17%) cases that did not report the specific mutation variant [10,14,15,27]. Our specific mutation, c.553-3A>G, has not been reported in any previous literature to date. Our cases highlight the importance of using WES to confirm a diagnosis of AMACR deficiency, which is also supported by previous studies that adopted this diagnostic tool (Table 2) [7,9,11].

**Table 2:** Summary of known adult and pediatric cases of alpha-methylacyl-CoA racemase deficiency. * The present study; † variant of uncertain significance; ‡ heterozygous mutation; § homozygous pathogenic variant. AMACR: alpha-methylacyl-CoA racemase; cDNA: complementary DNA; WES: whole exome sequencing; FAB-MS: fast atom bombardment mass spectrometry; GC-MS: gas chromatography-mass spectrometry; PCR: polymerase chain reaction; SNP: single nucleotide polymorphism; VLCFA: very-long-chain fatty acid.

Author, year	N	Sex	Age (Y)	Diagnostic tool	Gene variant	Treatment
Isa et al. (2023)*	2	F, M	6, 11	WES	c.553-3A>G^†^	Cholic acid, fat-soluble vitamins
Wang et al. (2021) [2]	1	M	71	High-throughput sequencing of exons and their adjacent introns	c.710A>G^‡^	Oral ursodeoxycholic acid, bicyclol, spironolactone tablets, and vitamin A supplements
Alsalamah et al. (2021) [7]	3	M, F, F	2, 12, 13	WES with targeted segregation analysis	c.877T>C^§^	-
Haugarvoll et al. (2013) [9]	2	M, F	30, 33	Homozygosity mapping, exome sequencing, whole genome SNP genotyping, and WES	c.367G>A^§^	Lamotrigine, restricted phytanic, and pristanic acid diet
Saxena et al. (2022) [10]	1	M	4.5 months	Exome sequencing	-^‡^	-
Setchell et al. (2003) [11]	1	F	2.5 months	FAB-MS of bile acids in urine and DNA analysis	c.154T>C^§^	Cholic acid
Gündüz et al. (2019) [12]	1	M	10 months	Next-generation sequencing technique and VLCFA analysis	c.596G>A^§^	-
Veldhoven et al. (2001) [14]	1	F	1 week	FAB-MS of urine	-	-
Stewart et al. (2011) [15]	1	M	45	Urine and bile analysis	-	Plasma exchange and dietary restriction of fatty acids
Thompson et al. (2009) [16]	1	F	57	Sequence analysis of AMACR cDNA, bile acid analysis in urine and plasma	c.154T>C^§^	Restricted phytanic and pristanic acid diet
Tanti et al. (2022) [19]	1	F	7^th^ decade	Enrolled in the 100,000 genome project	c.154T>C^§^	Lacosamide, low pristanic and phytanic acid diet
Ferdinandusse et al. (2000) [20]	2	M, F	44, 48	Sequence analysis of AMACR cDNA in Escherichia coli	c.154T>C^§^	-
	1	M	18 months	Sequence analysis of AMACR cDNA in Escherichia coli	c.320T>C^§^	-
Mclean et al. (2002) [21]	1	M	44	Fatty acid and bile acid analysis in serum and fibroblast studies	c.154T>C^§^	-
Clarke et al. (2004) [22]	1	F	53	Cultured fibroblasts by enzyme measurements and sequence analysis of AMACR cDNA	c.154T>C^§^	Low pristanate and phytanate diet
Smith et al. (2010) [23]	1	M	45	VLCFA analysis in serum by GC-MS and PCR amplification of AMACR gene	c.154T>C^§^	Dietary pristanic acid restriction
Dick et al. (2011) [24]	1	M	58	VLCFA analysis and plasma bile acid analysis	c.154T>C^§^	-
Krett et al. (2021) [25]	1	M	6^th^ decade	WES (but for myopathies)	c.154T>C^§^	-
Kapina et al. (2010) [26]	1	M	23	Biochemical analysis of cultured skin fibroblasts, immunofluorescence microscopy analysis using antibodies against catalase in skin fibroblasts, molecular analysis of AMACR gene	c.559G>A^§^	Dietary restriction of phytanic and pristanic acid
Verhagen et al. (2012) [27]	1	M	1^st^ decade	SNP array analysis, GC-MS of VLCFA, branched-chain fatty acids, and bile acid intermediates	Deletion of chromosome 5p13.3^§^	-

The primary treatment for BASD type 4 is cholic acid. If given with chenodeoxycholic acid or ursodeoxycholic acid, it shows reduced efficacy. In addition, no adverse effects arise even with multiple years of treatment [28]. Cholic acid is given at a dose of 10-15 mg/kg body weight per day and is titrated in response to the change in urine metabolites. Exogenous administration of cholic acid aims to replenish the reduced amounts of bile acids. Subsequently causing a decrease in the abnormal synthesis of endogenous bile acids through a negative feedback mechanism. This hinders further damage done by the toxic intermediates [18,28,29]. In a case by Setchell et al., chronic supplementation of cholic acid reverted liver enzymes back to normal and prevented future progression of symptoms [11]. Since malabsorption of these vitamins is a feature of this disease, supplementation of the deficient fat-soluble vitamins is a crucial pillar in the treatment strategy [2,11,14].

Another treatment modality is leveraging diet to exclude sources of pristanic and phytanic acid. This may offer a long-term solution to the management of this disease; however, the efficacy of this has not been established extensively in the literature [16]. In one case of adult-onset AMACR deficiency, following a 16-month dietary intervention involving restricted pristanic acid resulted in clinical improvement in encephalopathic episodes, language, and gait [23]. Compliance and tolerance with the diet can be a potential issue [7,21]. Diet efficacy can be potentially assessed through multimodal and electrophysiological monitoring of the retina [7]. However, further studies need to be conducted to conclusively link this association. In our case, both patients were advised to supplement with cholic acid. Therefore, we expect a positive response, but follow-up is needed to properly document the efficacy. In addition to this, our patients were supplemented with fat-soluble vitamins and referred to a dietician for further optimization of their diets.

## Conclusions

In this report, we highlight the subtle presentation of two siblings with a novel mutation in the AMACR gene as well as autoimmune antibody positivity. Initial presentations of this disorder continue to vary case by case and paint an ambiguous clinical presentation that can easily be missed. Patients presenting with isolated elevated LFTs and unexplained cholestatic symptoms should raise suspicion for BASD. WES is becoming an increasingly effective and confirmatory test to diagnose BASD. In our case, WES conclusively diagnosed the two patients, while urine testing was inconclusive and did not support the diagnosis. This reiterates the need to keep BASD in a differential even if urine testing is normal. Cholic acid continues to be the mainstay of treatment; however, more studies are needed to solidify its role in the treatment of BASD type 4. More case reports would benefit the growing clinical image of AMACR deficiency presentation.
